# A New Perspective on the Treatment of Alzheimer's Disease and Sleep Deprivation-Related Consequences: Can Curcumin Help?

**DOI:** 10.1155/2022/6168199

**Published:** 2022-01-12

**Authors:** Esra Küpeli Akkol, Hilal Bardakcı, Çiğdem Yücel, Gökçe Şeker Karatoprak, Büşra Karpuz, Haroon Khan

**Affiliations:** ^1^Department of Pharmacognosy, Faculty of Pharmacy, Gazi University, Etiler, 06330 Ankara, Turkey; ^2^Department of Pharmacognosy, Faculty of Pharmacy, Acibadem Mehmet Ali Aydınlar University, 34752 Istanbul, Turkey; ^3^Department of Pharmaceutical Technology, Faculty of Pharmacy, Erciyes University, 38039 Kayseri, Turkey; ^4^Department of Pharmacognosy, Faculty of Pharmacy, Erciyes University, 38039 Kayseri, Turkey; ^5^Department of Pharmacognosy, Faculty of Pharmacy, Başkent University, Etimesgut, 06790 Ankara, Turkey; ^6^Department of Pharmacy, Abdul Wali Khan University, Mardan 23200, Pakistan

## Abstract

Sleep disturbances, as well as sleep-wake rhythm disorders, are characteristic symptoms of Alzheimer's disease (AD) that may head the other clinical signs of this neurodegenerative disease. Age-related structural and physiological changes in the brain lead to changes in sleep patterns. Conditions such as AD affect the cerebral cortex, basal forebrain, locus coeruleus, and the hypothalamus, thus changing the sleep-wake cycle. Sleep disorders likewise adversely affect the course of the disease. Since the sleep quality is important for the proper functioning of the memory, impaired sleep is associated with problems in the related areas of the brain that play a key role in learning and memory functions. In addition to synthetic drugs, utilization of medicinal plants has become popular in the treatment of neurological diseases. Curcuminoids, which are in a diarylheptanoid structure, are the main components of turmeric. Amongst them, curcumin has multiple applications in treatment regimens of various diseases such as cardiovascular diseases, obesity, cancer, inflammatory diseases, and aging. Besides, curcumin has been reported to be effective in different types of neurodegenerative diseases. Scientific studies exclusively showed that curcumin leads significant improvements in the pathological process of AD. Yet, its low solubility hence low bioavailability is the main therapeutic limitation of curcumin. Although previous studies have focused different types of advanced nanoformulations of curcumin, new approaches are needed to solve the solubility problem. This review summarizes the available scientific data, as reported by the most recent studies describing the utilization of curcumin in the treatment of AD and sleep deprivation-related consequences.

## 1. Introduction

Plants have a long history of use as a remedy for numerous health problems. When we look at the traditional treatments, *Curcuma longa* L. (Zingiberaceae), commonly known as turmeric, comes to the fore. Turmeric has been reported to be used especially in Ayurveda and has religious importance in Hinduism [1]. Moreover, it is mostly used as a spice and a food-coloring agent in Southeastern Asian cuisine [2]. Curcumin, a natural compound isolated from *C. longa*, has multiple applications in treatment of various diseases such as cardiovascular diseases [3], liver disease [4, 5], obesity [6, 7], cancer [8], inflammatory diseases [9, 10], and aging [11, 12].

Besides these applications and activities, curcumin has been reported to be effective in many neurodegenerative diseases. Alzheimer's disease (AD) is one of the neurodegenerative diseases and represents a high percentage of human deaths worldwide. Based on the presented epidemiological data, a group of authorities estimated that 24.3 million people have AD today, with 4.6 million new cases of AD every year. The number of people affected will double every 20 years to 81.1 million by 2040. Studies have shown that curcumin can lead to many improvements in the pathological process of AD [13]. Its mechanisms of action can be classified as (I) sustaining homeostasis of the inflammatory system, (II) increasing the clearance of toxic substances from the brain, (III) scavenging free radicals and inducing antioxidant elements, and (IV) binding and limiting aggregation of misfolded proteins [1]. Although curcumin has a wide range of therapeutic actions, its low solubility and bioavailability limits its utilization in the clinic. Novel pharmaceutical formulations needed to be developed in order to solve improve its pharmacokinetic parameters [13].

In addition to the effects of curcumin on neurodegenerative diseases, especially AD, it has been reported that curcumin can be beneficial in the management of sleep deprivation (SD) and SD-induced neurological consequences as well [14]. SD has shown to have negative influence on the physical, psychological, and neurological functions. Unfortunately, problems of SD are increasing in public nowadays [15]. The effects of curcumin on the medial prefrontal cortex (mPFC) [16], the protective potential on the dendritic trees from shortening and shedding induced by chronic SD [14], and the effects of SD-induced memory impairments have been demonstrated by *in vivo* studies [17].

New alternative treatments for neurological disorders caused by AD or SD, as well as better formulations to overcome curcumin's therapeutic limitations, are still being researched. Recent review studies on this area are also very important and can provide researchers with new perspectives.

## 2. Assessment of Sleep and Cognitive Impairment

The investigation of sleep and rest problems requests a conduction of a pile of detailed strategic diagnostic techniques. Subjective and objective methods are the mostly preferred methods. Recording scales of manner and sleep is amongst the subjective evaluation techniques. Polysomnography and actigraphy are the primarily chosen objective methods.

Regardless of the benefits of subjective techniques like accessibility and diminished expenses, these may prompt tremendous rates of false-positive and false-negative results meddling the clinical evaluation. Several investigations have demonstrated the inconsistency between the data acquired by subjective and objective techniques. For instance, individuals with mild cognitive impairment (MCI) exhibited more prominent misperception of their sleep beginning time than the analogous healthy ones [18].

Actigraphy is a trustworthy technique to evaluate the sleep quality and nature, notwithstanding being less tender than polysomnography. The utilization of this technique is reasonable in circadian cycle assessments and diagnosing the periodic limb movement disorder. The benefits of this objective method are its being cost effective, ease of application, lower degrees of confliction with the sleep quality, and the chance of mapping the whole motor activity of the individuals for longer intervals [19].

Nonetheless, polysomnography gives more exact results comparing with the actigraphy in recognition of various sleep disorders (for instance, breathing problems, recurring limb movement, and rapid eye movement sleep (REM) behavioral disorder), and changes in sleep characteristics as well. Due to the higher charges and the need for keeping the patients in a sleep laboratory, its practice is more challenging. In addition, sleep attitudes especially in grown-ups with higher recurrence of circadian rhythm alterations and daytime snoozes could not be evaluated by polysomnography.

## 3. Sleep Changes in Mild Cognitive Impairment

**Figure 1:**
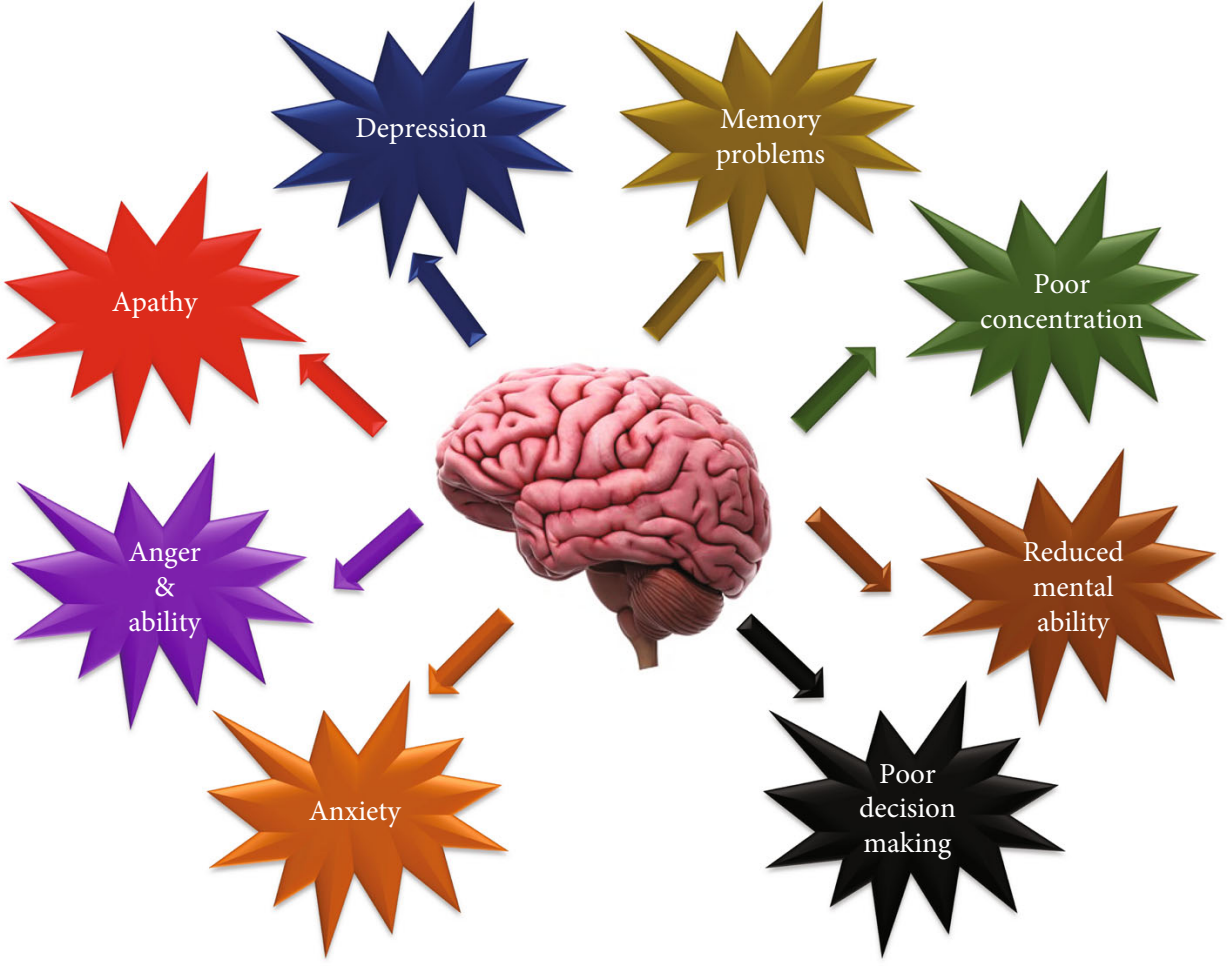
The symptoms of mild cognitive impairment (MCI)

Memory deterioration besides the age and education without presenting any critical functional alterations is the particular feature of MCI. Scientists studying cognitive functions grouped MCI as amnestic and nonamnestic MCI and classified each group into specific and multiple domains according to neuropsychological assessments. This grouping is crucial because individuals with multiple-domain amnestic MCI show a higher rate of progress dementia [20]. The symptoms of MCI are presented in Figure 1.

Findings from large-population studies associate MCI with higher rates of sleeping disorders compared with healthy people at the same age. The currency of sleep disorders in patients with MCI changes from 8.8% to 59% [21]. Furthermore, basic sleep disturbances, like breathing problems, recurring limb movement, and REM sleep behavioral disorders are frequently seen in MCI [22].

The correlation between the sleep disturbances and higher incidences of progression of MCI to AD has been revealed by prospective cohort studies [23]. A meta-analysis of researches comparing both objective sleep measures of patients with amnesic and nonamnesic MCI along with the healthy grown-ups revealed that both MCI subtypes showed a greater cyclic alternating pattern, which is an expression of sleep instability and elongated sleep latency along with the decreased sleep efficiency and total sleep time. Patients with amnesic MCI expressed a reduced arousal index and total sleep time compared with the nonamnesic MCI patients. No difference was seen between the assessed groups in terms of wake time after sleep onset, slow-wave sleep, REM duration, REM (%), and recurrent limb movement syndrome [19]. Still, patients with MCI demonstrate elevated night-time attitudes [24].

Cognitive performance with amnestic MCI and healthy individuals were compared using neuropsychological tests. Initially, neuropsychological and polysomnographic assessments of the patients were performed former and latter sleep for two days. Word pair recall, fact recognition, and object promising tests were performed, and the control group exhibited increased memory performance after sleep. On the contrary, memory performance did not recover in amnestic MCI patients; even the word pair recalls test showed the memory relapse. Nonamnestic MCI patients also suffer from sleep disorders. Memory dysfunction and elongated wake time after sleep onset with another dysfunctions (attention deficiency, response inhibition, concept forming, and critical thinking) have been related with the sleep discontinuity [25]. Researches related with sleep characteristics revealed that the MCI patients has decreased slow-wave sleep compared with healthy individuals [26, 27] and elevated fragmentation of non-REM sleep as well. Furthermore, ambivalent differences were realized in REM, wake time after sleep onset, REM duration, and sleep quality [27].

The evaluation of EEG power across non-REM and REM sleep likewise uncovered contrasts between patients with amnestic MCI and the healthy ones. Initially, alleviation in power of delta and theta EEG frequency bands was seen. Similar decrease has been seen in the fast spindle counts on frontal locations. The correlation between the decrease in delta and theta power along with a decreased cognitive performance latter sleep was discovered [27]. Besides, the REM span was diminished by 5% in patients with MCI. Prolonged REM duration rapidly enhanced the cognitive performance [28].

## 4. Sleep Changes in Alzheimer's Disease Dementia

**Figure 2:**
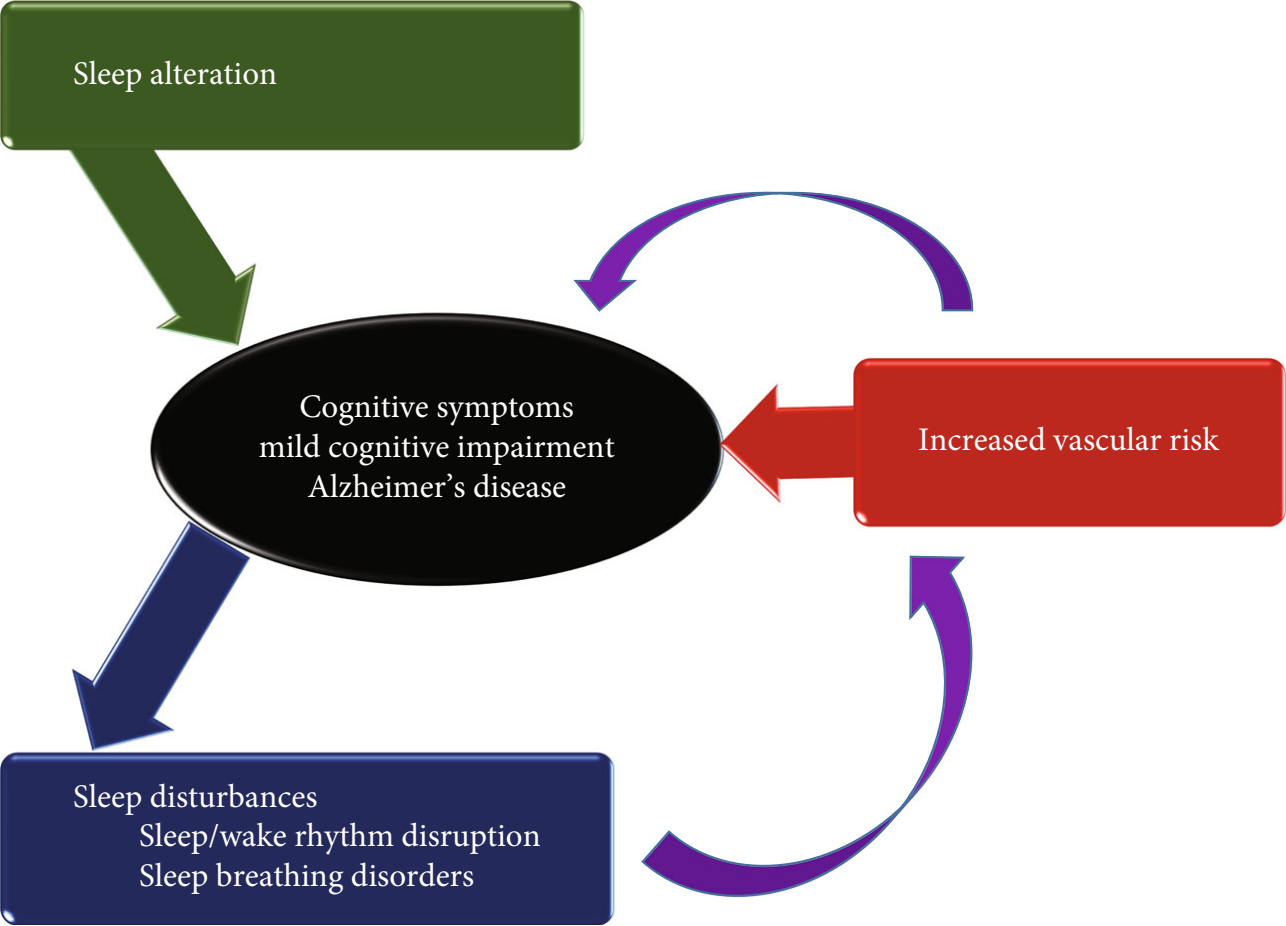
Possible interactions between sleep disorders and Alzheimer's disease.

In the 1980s and 1990s, electroencephalographic studies in AD dementia patients and healthy individuals afforded invaluable data about the sleep patterns and neuropsychiatric disorders, such as depression as well. AD dementia and healthy volunteers showed similar sleep disturbances [29]. However, the seriousness of the shifts was typically higher and the REM sleep had explicit differences. In AD dementia patients, sleep was much upset at night, attentiveness was progressed, and the number and span of sleep were disturbed a lot, resulting a shorter total sleep period with lower quality [30]. As a conclusion, the superior ratio of stage 1 and 2 sleep and the decreased ratio of slow-wave sleep were seen in the patients [31]. Possible interactions between sleep disorders and AD were shown in Figure 2.

In addition, AD dementia is identified with decreases of explicit highlights of the N2 (stage 2) light sleep period, for example, sleep spindles and K complexes, which become inadequately produced, with lower magnitudes and prevalences [31]. The significance of dementia has been known to be deteriorated due to these changes, and the development of disease makes discrimination of N1 (stage 1) and N2 difficult [30, 32], since sleep without noticeable phases seen in latter AD as indeterminate non-REM has been arranged [29, 31, 33].

During the progression process of AD, the cholinergic system begins to deteriorate, thus leading dementia-associated REM sleep changes. The degeneration of the basal forebrain in AD, induces decreased cortical activity during REM sleep [29, 31]. Similarly, quantitative EEG researches indicated greater power in theta and delta frequencies and diminished power in alpha and beta frequencies during AD patients' REM sleep. The total number of REM sleep episodes and REM sleep latency however remain intact [31].

Enhancing memories via sleep is thought to be due to the data reactivation and hippocampo-neocortical transfer related with the exact sleep brain oscillations such as slow oscillations, thalamocortical spindles, and hippocampal ripples during slow-wave sleep. Moreover, slow-wave sleep affects the hippocampus-dependent memories (declarative/episodic memories) whereas high amounts of REM sleep affect memories not depending on the hippocampus such as procedural learning related to fronto-striatal circuits. Although the mechanism is still hypothetical, sleep-related breathing disorders (SRBD) have an impact on neuronal dysfunction through hypoxia or sleep disruption or might assist the emergence or worsening of vascular and/or neurodegenerative brainstem lesions. In addition, during REM sleep, acetylcholine levels are boosted which is a critical factor in consolidation of procedural memories. The degeneration of the cholinergic system aids the symptomatic expression of AD [29].

Initial works that assessed the sleep scheme in AD patients were conducted without classification by severity. Nonetheless, advanced researches have been performed to characterize sleep in patients with mild-stage AD dementia as well. Predominantly, principal alteration in sleep/wake diagrams displayed was the considerable interference of wakefulness during sleep [30].

## 5. Sleep Disorders as Risk Factors for Alzheimer's Disease

In the 20th century, it was claimed that the sleep disruptions might serve as a contributing factor to dementia progression and the principal hypothesis was initially that these disorders emerged in tandem as the ageing process. However, this theory shifted after several prospective cohort analyses of dementia patients with history of sleep problems have greater chance of developing AD than those without sleep problems. One of the most recent meta-analyses approved the possible contribution of poor sleep and sleep disorders as a prognosticator of AD.

Recent prospective cohort studies conducted around 2000s revealed that disturbances and disorders related with sleep might be related with the elevated risk of cognitive impairment and dementia. Those studies revealed various forms of sleep disturbances and generally found them to be associated with dementia progression, for instance, the connection between insomnia [34], elevated daytime sleepiness [35, 36], elongated sleep periods [37, 38], shorter sleep period [37, 39, 40], and arising of cognitive impairment and/or dementia. As a summary, both short and long sleep durations might cause cognitive impairment/dementia [38, 39].

Participants were assessed by utilizing subjective methods in terms of sleep characteristics or cognitive appraisal in the beginning yet revealing the linkage amongst sleep disorders and cognitive functioning [35, 41]. Later (early 2000s), cohort studies with an adequate number of participants afforded more accurate data on dementia diagnosis by using standardized scales to evaluate sleep, though studies which were not tracked less than five years avoided respectable data [42].

In 2010, a literature was published revealing the relationship between the risks of cognitive impairment and sleeping more than nine hours per night after 10 and 14 years of monitoring patients. On the other hand, the use of telephone as a tool for interpretation of cognitive functions resulted in the questioning of this study [43].

**Figure 3:**
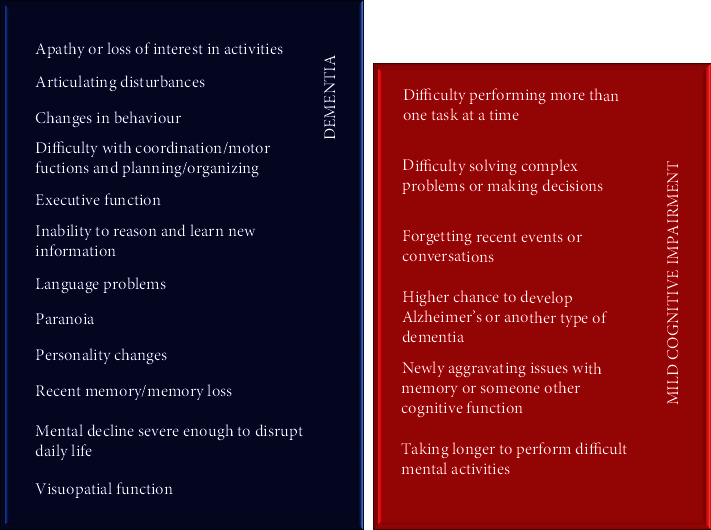
Symptoms of dementia and MCI

Tranah et al. demonstrated the strategical drawbacks in previously published studies in their detailed study about the evaluation of sleep and daily activities with wrist actigraphy conducted on 1,282 healthy women [44]. Attendants were exposed to a neuropsychological battery and dementia tests after a 4.9 years of monitoring. It was concluded that deteriorated circadian rhythms increased the dementia and MCI incidence. Along with the standardized sleep screening tools, more-detailed wrist actigraphy techniques were used in advanced studies [45]. Comparison of symptoms of dementia and MCI was shown in Figure 3.

Yaffe and colleagues demonstrated that healthy women with sleep-disordered breathing problems were more susceptible to develop MCI or dementia than women without sleep problems by using [46]. This claim was verified by monitoring a high number of participants for longer periods. Furthermore, Pase and colleagues discovered that the decrease in REM sleep instead of non-REM sleep is also related with dementia [47].

There were no such studies continued more than 15 years to evaluate the association of sleep disorders and cognitive performances even though the arise and amelioration of AD takes 10–15 years. The earliest research which lasted for 22.5 years was conducted on Finnish patients at least 65 years old [37]. Diminished cognitive performance was obtained on patients with short (<7 hours/day) and long (>8 hours/day) durations of sleep and insufficient sleep quality. Pase and colleagues performed resembling analysis confirming results with Virta and colleagues [47].

Supplementary data were obtained by several follow-ups. For instance, the connection between the sleep disorders and cognitive impairment non-AD of vascular origin is exhibited [36]. Moreover, early associations of sex, cognitive performances, and various kinds of sleep disruptions were declared by Potvin and colleagues [39].

Subsequent to the demonstration of the significant association between cognitive performance and sleep quality, epidemiological investigations were performed. Almondes and colleagues explained that individuals with the insomnia background showed higher risk of developing dementia (RR = 1.53; 95%CI 1.07–2.18) [48]. In a genuine research series including 27 studies with 69,216 participants, sleep disorders except insomnia likewise showed a higher risk of developing AD (1.55 (95%CI: 1.25–1.93)), cognitive impairment (1.65 (95%CI: 1.45–1.86)), and preclinical AD (3.78 (95%CI: 2.27–6.30)) [49]. In addition, meta analyses were performed for the diagnostic role of sleep disorders in the frequency of cognitive impairments. Shi and colleagues classified sleep disorders as insomnia, breathing problems, longer daytime sleepiness, associated movement disturbances etc. and confront them with the various types of diminished cognitive performance one by one using 18 papers between the previously published 12,926 papers. According to the findings, insomnia is associated with AD, and sleep-related respiratory difficulties are associated with all types of dementia, including AD and vascular dementia. [40]. However, further researches should be conducted for more significant claims.

Diagnosis of AD is challenging, though biomarkers are used in the prediction of the disease [50]. In this case, polysomnography and lumbar puncture were used to investigate the relation between sleep and AD. The association between sleep-disturbed breathing and AD was revealed by evaluation of cerebrospinal fluid biomarkers [51]. Further studies appraising PET with amyloid and tau imaging are ongoing.

## 6. Potential Mechanisms Linking Sleep Disorders and AD Risk

Although clinical data revealed the association amongst sleep characteristics and AD, in order to expose the underlying mechanisms, further in vivo and clinical studies were conducted. F-Fluorodeoxyglucose (FDG-PET), a biomarker showing the metabolic activity of brain, is calculated during awake and sleep states. The levels were found to be identical in REM sleep and being awake; however, the cerebral metabolic activity was diminished considerably (around 40%) at the time slow-wave sleep [52, 53]. This impairment was claimed to be located in subcortical and cortical regions and might be due to the neurons' overpolarized silent state during slow-wave sleep [53]. Discharge of amyloid-*β* (A*β*) in the interstitial fluid involves the synaptic activity [52], which is also showed by some other experiments [54, 55]. Longer wakefulness leads to higher A*β* concentration and further escalation is seen during acute sleep deprivation. Alleviated A*β* grades develop in sleep exclusively during slow-wave sleep on the contrary [55].

Glial cells have a significant role in toxic metabolite elimination of the brain and called as the glymphatic system and functions analogous to the lymphatic system. Experiments with rodents revealed that CSF is moved to the periarterial spaces of the wider cerebral arteries from the subarachnoid areas, running along them as they branch into penetrating arteries. The CSF is driven from the perivascular spaces into the brain interstitium supported on the end feet of the astrocyte by AQP4 transporters. A similar system is seen from the interstitium to the perivenous spaces; CSF is transported in the subarachnoid space. Various pathways produce a connective bulk flow of interstitial fluid (ISF) from the periarterial to the perivenous spaces via the interstitium facilitating the washout of toxic metabolites accumulated in the brain like A*β* and tau protein [56, 57]. An investigation conducted in 2012 uncovered that a 60% extension in the interstitium happens during sleep that aids convective trade among CSF and ISF, advancing an augmented A*β* clearance during sleep [58]. In conclusion, an alleviated convective transfer between ISF and CSF leading to diminished A*β* clearance could be caused by sleep disorders.

In total, both possible results of disturbed sleep periods (an expanded cerebral performance with augmented A*β* levels and diminished working of the glymphatic exchanges with diminished A*β* elimination) might prompt higher levels of soluble A*β* in the ISF, which is related with amyloid degradation [59]. The precuneus, lateral parietal, and medial prefrontal brain regions are involved in the default mode system, which is progressively dynamic in the resting periods, and the system with the highest neuronal activity [60]. Moreover, this network is the most easily affected region to A*β* assembly in the initial phases of AD.

Circadian rhythm disturbances are one of the potential risk factors in neurodegeneration and AD. Studies demonstrated that hippocampal-dependent learning regulation, diminished hippocampal neurogenesis, impaired learning and memory, and higher biomarkers related with inflammatory conditions were emerged after circadian rhythm abnormalities [61]. Later, Zhou and colleagues discovered that the suprachiasmatic nucleus degeneration is the elemental cause of the circadian rhythm abnormalities in individuals with AD and the sundown syndrome [62].

Furthermore, neuronal oxidative stress, blood-brain barrier degeneration, and disruption in hippocampal neurogenesis are some of the other features caused by sleep disorders [63].

Amyloid plaques are composed in regions of the brain controlling sleep, which further disturb sleep. Similarly, it leads more behavioral symptoms with AD dementia, such as changes in lifestyle, diminished exposure to daylight, utilization of polypharmacy, and decreased sleep quality. Another reason for sleep problems that could meddle legitimately in the pathogenesis of AD is sleep-disturbed breathing, through hypoxia and inflammatory cascades.

## 7. Treatment Considerations

The treatment should not be initiated according to the symptoms before the diagnosis of the etiology, because symptoms and diseases might interfere or cover existing disease such as delirium and dementia in geriatric individuals. For instance, pharmaceuticals used in the treatment of acute urinary tract infections in dementia patients with nocturnal agitation might lead to sleep disruptions. Moreover, early phases of delirium in geriatric patients might be related with poor nutrition, physical restraints, bladder catheter utilizations, polypharmacy, or iatrogenic conditions [64]. Herein, correct differential diagnosis plays an important role.

Sleep disorders should be verified by polysomnography before the treatment in case of the absence of any medical comorbidity. In case prior behavioral characteristics is seen in the evenings or nights and any family member could witness the situation, restless legs syndrome (RLS) should be examined. Neuroimaging examinations displayed the decreased striatal dopamine levels on positron emission tomography in wandering AD patients with RLS [65]. In case of RLS, dopamine agonist medications might be preferred. Nevertheless, the diagnosis should be accurate and should be tested by measuring the serum ferritin levels which is lower in patients with dementia and RLS [66].

Nocturnal behavioral characteristics must be evaluated if the diagnosis of sleep disorders fails. Triazolam (benzodiazepine type medicine) was tested in a small-scale clinical trial and showed favorable results [67]. The choice of GABA A agonists in AD treatment is debatable; there are studies with controversial results [68]. The use of zolpidem in the improvement of sleep disturbances, insomnia, and wandering in patients with dementia and Parkinson's disease was found to be effective in earlier studies [69–71]. However, today, many geriatricians are not willing to use sedative-hypnotics. Mainly, the sedative-hypnotics with long elimination half-lives are especially not preferred because they leading to several adverse effects [72]. Nocturnal psychotropic could be diminished without a problem [73, 74]. Staffing issues have an essential place in PRN dosing as do patient-related factors [75] and a long-standing literature linking psychotropic and falls and hip fractures [76, 77]. Zolpidem with a clearance half-life of 2.5 h might drive patients to falling [78]. Whereas, there is not any precise evidence showing the association among the sedative-hypnotic use and falls [76, 77, 79, 80]. The general behavior of individuals with sleep problems is to use sedative-hypnotics; however, they often awaken during night aside from the medication. Hence, falls might be related with poor sleep instead of medications. Brassington and colleagues revealed that sleep interruptions are the cause of falls in older communities, proving this hypothesis. [81].

Prescription of cholinesterase inhibitors (donepezil, galantamine, rivastigmine, etc.) is frequently preferred in the treatment of AD, and those also affect sleep. There is a connection between REM sleep and the cholinergic system; hence, longer REM durations might be seen in patients who are using cholinesterase inhibitors. This hypothesis is confirmed with other studies; nondemented patients aged > 65 used donepezil and healthy individuals that used galantamine showed counterpart changes in REM durations [82, 83]. The density of eye movements during REM in healthy individuals has been increased by use of rivastigmine [84]. It is uncertain whether such polysomnographic impacts may likewise happen in AD. One may guess that, given the significance for the REM period and learning and memory, increments in REM might be identified.

There are case reports of striking dreaming and nightmares in AD patients getting cholinesterase inhibitors supporting this claim [85, 86]. Regardless of whether such encounters are additionally reflected in the frequently announced ones, the everyday unfavorable impact of sleep deprivation is hazy, yet would show up in any event conceivable. In this context, the frequency of insomnia was examined by using various doses of donepezil; however, both rivastigmine and galantamine were found to have lower effects on sleep [87–90]. Data from pharmacosurveillance shows that donepezil use in AD is associated with more than twice the rate of sedative-hypnotic prescriptions when compared to AD patients who do not take donepezil. This adds to the growing body of evidence that cholinesterase inhibitors are likely to disrupt sleep in AD patients, despite their widespread use. [91]. Despite the fact that rivastigmine and galantamine do not disrupt sleep, there is limited evidence that cholinesterase inhibitors improve sleep in Alzheimer's patients. Against this backdrop of evidence suggesting that cholinesterase inhibitors may have limited utility as explicit medications for improving nighttime fomentation and sleep in Alzheimer's disease, a few reports recommend their use in the treatment of dream enactment behaviors associated with Lewy body dementia (LBD) [92, 93]. This information is persistent with frequent cortical cholinergic loss in LBD that is in some point, in excess of that seen in AD.

## 8. Chemistry of Curcumin

**Figure 4:**
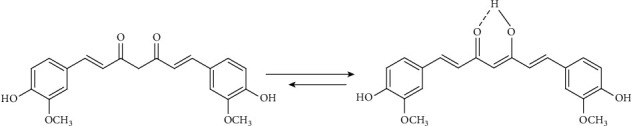
Curcumin: diketone and keto-enol form equilibrium

The structure of the compounds that occur in a mixture called curcuminoids, which are the main components of turmeric, usually constitutes about 1–6% of the plant by dry weight and is in the diarylheptanoid structure. Three major compounds form curcuminoids: curcumin (1E,6E)-1,7-bis(4-hydroxy-3-methoxyphenyl)-1,6-heptadiene-3,5-dion, demethoxycurcumin, and bisdemethoxy curcumin [94]. Its chemical formula is C_21_H_20_O_6_ and the molecular weight is 368.38 g/mol. Two aromatic ring systems containing o-methoxy phenolic groups, connected by a linkage with seven carbon atoms including an *α*, *β*-unsaturated *β*-diketone moiety, form as three chemical entities in the curcumin structure [95]. The transfer of intramolecular hydrogen atoms between the *β*-diketone group leads to the presence of the enol-tautomer form and exhibits better solubility in dimethyl sulfoxide and chloroform, mild solubility in methanol, and limited solubility in water. In acidic and neutral conditions, the major component is in the diketone form, acting as a proton donor, while in the alkaline state, the keto-enol form acts as the electron donor as the main component (Figure 4) [96]. Curcumin has three water-ionizable protons: enolic protons with an approximate 8.5 pKa and two phenolic protons with 10–10.5 pKa (in mixed alcohol/water solvent) [97]. Curcumin shows spectrophotometrically strong absorption ranging from 408 to 434 nm [96].

**Figure 5:**
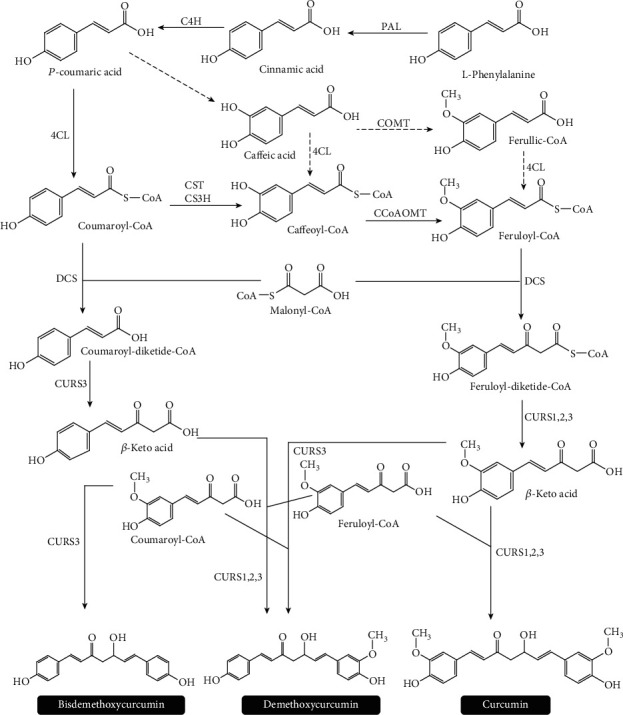
Biosynthetic pathway of curcuminoid [98]

The first studies on the biosynthesis of curcuminoids illuminated that they were synthesized from the phenylpropanoid pathway. The biosynthetic pathway of curcumin starts with phenylalanine and involves 2 units of cinnamic acid and one central carbon from malonic acid and the following addition of functional groups on the aromatic rings. Phenylalanine ammonia lyase is the first enzyme in the biosynthesis pathway. Curcumin synthase with three isomers (CURS1,2,3) and diketide-CoA synthase (DCS) and polyketide synthase enzymes were isolated from the *C. longa*. DCS is the enzyme that forms the feruloyl-diketide-CoA by catalyzing the reaction of feruloyl-CoA with malonyl-CoA. Curcumin synthase takes place in the decarboxylative condensation reaction in the biosynthesis of curcuminoids. Both DCS and CURS1, 2, and 3 have starting substrates to be converted into one of the curcuminoids. The feruloyl-CoA starter unit converts to curcumin via CURS. CURS also catalyzes converting of p-coumaroyldiketide-CoA and feruloyl-CoA to give demethoxycurcumin. Enzymes *p*-coumaroyl shikimate transferase, *p*-coumaroyl quinate transferase, caffeic acid *O*-methyltransferase, and caffeoyl-CoA *O*-methyl-transferase were also involved in the curcuminoid biosynthetic pathway (Figure 5) [98].

## 9. New Delivery Systems Used to Increase Curcumin Bioavailability

Nanosized delivery systems were investigated which supply superior opportunities in the management of central nervous system (CNS) diseases such as AD that can be modified with targeting agents to bind receptors or transporters expressed at the blood-brain barrier (BBB), thereby increasing CNS selectivity and permeability [99]. It has been known that natural bioactive compounds such as curcumin are effective in treatment for many years, but overcoming their low bioavailability is possible with the use of nanocarrier systems [100, 101]. In previous studies, nanosized delivery systems were developed with curcumin that are widely used to achieve better therapeutic outcome [102, 103]. It has been reported that curcumin possesses neuroprotective activity specifically on neurodegenerative diseases like AD which progresses with the accumulation of *β*-amyloid peptide in senile plaques, which is the most important histopathological symptom of the disease. Curcumin has been shown to have the possibility of slowing the progress of AD by reducing amyloid *β*. At the same time, curcumin has taken its place in many studies to be used in the treatment of AD as a prominent compound with its antioxidant effect [104, 105]. The use of nanotechnology in drug delivery systems improves the bioavailability of drugs in biological systems [106]. Because of curcumin's rapid systemic elimination, many nanosized delivery systems were used to increase curcumin circulation time in the body for the penetration of curcumin to different regions of the brain [107]. It was proven that nanoformulated curcumin decreased the *in vitro* and *in vivo* A*β* generation and also inhibited the aggregation of A*β* deposits [108].

Liposomes (LPs) are spherical bilayered phospholipid, micro- or nanosized, biocompatible, and nontoxic because of the similarity to biological membranes and entrap both hydrophobic and hydrophilic compounds. LPs have been used in the pharmaceutical field for many years as a nanocarrier to serve the therapeutic potential of active agents as curcumin in the treatment of AD [99, 109]. Lazar et. al. designed biocompatible curcumin-conjugated nanoliposomes (CnLs) for targeting amyloid deposits. Owing to be covalently linked to the surface of the CnLs, curcumin could interact directly with the amyloid deposits. In vitro studies were performed, and uniform, stable, and nontoxic CnLs were found; the secretion of A*β* was regulated and A*β*-induced toxicity was prevented. CnLs strongly labeled A*β* deposits on postmortem brain tissue of AD patients and transgenic mice overexpressing AD-related human mutations (APPxPS1 mice) [110].

Another different liposomal curcumin formulation was prepared by Taylor et al. to investigate the aggregation of the amyloid-*β*1-42 (A*β*1-42) peptide in vitro. The authors formulated different nanosized LPs (nLPs) incorporating or decorated (by click chemistry) with curcumin, the curcumin derivative, lipid ligand phosphatidic acid, cardiolipin, or GM1. All prepared nLPs with curcumin or its derivative were able to prevent the formation of fibril and/or oligomeric A*β*. The click-curcumin type, one of the three liposomes, was the most effective and nLPs with lipid ligands only inhibited A*β* fibril and oligomer formation at a very high LP/peptide ratio. [111].

Mourtas et al. developed and characterized multifunctional nLPs, which are containing curcumin derivate and decorated with mAbs (anti-transferrin Ab) as a transport mediator in LPs. Curcumin-derivative nLPs with and without anti-TrF showed a high affinity for the senile plaques on postmortem brain samples of AD patients, and both nLPs displayed to reduce A*β* (1–42) peptide aggregation using the thioflavin assay. Nevertheless, the mAbs-decorated curcumin-derivative nLPs significantly improved the intake by the BBB in vitro cellular model [112].

In another study by Mourtas and coworkers, curcumin-conjugated nLPs were formulated using different methods: the conventional method and the click chemistry technique. The nLPs containing the curcumin derivative showed high affinity for A*β* (1-42) fibrils and significant amounts of labeled A*β* deposits in postmortem brain tissue of AD patients. Whereby, nonplanar curcumin was not connected to A*β* (1–42) due to the emergence of multivalent interactions. In vivo injection into the hippocampus and the neocortex of transgenic mice has shown that overexpressing AD-associated human mutations (APP/PS1mice) and developing a large number of A*β* deposits showed that curcumin-conjugated nLPs could specifically stain A*β* deposits. Finally, these curcumin-conjugated nLPs may be preferred in diagnosis or treatment of AD [113].

**Figure 6:**
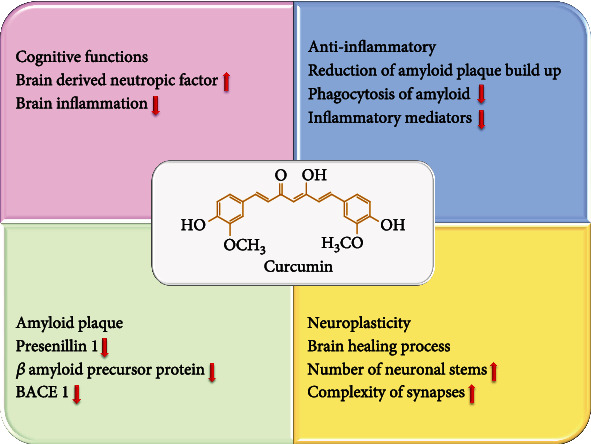
The mechanism of action of curcumin nanoformulations in Alzheimer's disease

The mechanisms of action of curcumin in AD have been proven by many studies (Figure 6) [114–117].

Other liposomal formulations were formulated with curcumin and conjugated with an anti-transferrin receptor antibody or cell-penetrating TAT peptide. The increased cellular uptake and higher permeability across a BBB model were found in comparison to nondecorated LPs [108, 118].

Exosomes are natural nanovesicles that are released into the environment by almost all cells, whose membranes are surrounded by a phospholipid bilayer (phospholipid and cholesterol), and their sizes vary between 30 and 100 nanometers. They contain various nucleic acids, lipids, and proteins. Exosomes, a natural nanopharmaceutical with the potential to be used in diagnosis and treatment in medicine, have many desirable features of the ideal drug delivery system. Exosomes are cell-derived vesicles with therapeutic potential and high biocompatibility. Proposing a new concept of exosome-like liposomes for drug delivery, Fernandes and his colleagues selected a model molecule of curcumin and examined curcumin exo-liposome effectiveness against oxidative stress and in decreasing *β*-amyloid accumulation in the treatment of AD. It was reported that new-type liposomes produced with high encapsulation efficiency were noncytotoxic on SH-SY5Y neuronal cells and had neuroprotection potential. Curcumin-loaded exo-liposomes were internalized, and the curcumin was released inside the cells. This innovative transporter is a new and effective approach to deliver drugs to the brain, as it is stable, retains the charge, and is retained by neuronal cells [119].

Nanoparticles (NPs) are named colloidal carriers in which drugs are encapsulated or adsorbed to the surface. These drug delivery systems provide sustained and controlled drug delivery across BBB and increased circulation lifetime. Many modifications with polyethylene glycol (PEG), peptides, polymers, or antibodies can be used by either adsorption or chemical binding on the surface of NPs to improve drug delivery. Recent studies indicate that delivery of NPs, especially modified NPs, is more effective in the brain [99, 120].

Mathew et al. prepared amyloid-binding aptamer (NN2) conjugated to poly(lactic-co-glycolic acid) (PLGA)-coated curcumin NPs to bind to the amyloid plaques and prevent their aggregation. They proposed to utilize the potential of aptamers as novel therapeutic tools that efficiently attach to the amyloid fibrils and to evaluate effectiveness of curcumin-encapsulated PLGA NPs against amyloid plaques. It was reported that NN2–curcumin–PLGA NPs attached with the amyloid proteins and disaggregation of amyloid proteins could be observed at formulated NPs with and without aptamer. It was understood that the antiamyloid activity of the attached aptamer did not change from SEM images. However, the protein aggregate size was decreased when aptamer-conjugated curcumin–PLGA NPs and amyloid aggregates were incubated together [121].

In another study, Cheng et al. prepared a highly stable nanocurcumin using a polyethylene glycol-polylactic acid (PEG-PLA) coblock polymer and polyvinylpyrrolidone (PVP). This new formulation effectively transported the curcumin across BBB with around a sixfold increase in the mean residence time in the brain compared to nonformulated curcumin. Also, better cue memory was observed in Tg2576 mice in contextual fear conditioning tests treated with nanocurcumin [122]. Fan and coworkers designed curcumin-loaded PLGA-PEG NPs conjugated with B6 peptide and to evaluate the potential efficacy of PLGA-PEG-B6 containing curcumin in HT22 cells and APP/PS1 Al transgenic mice for the treatment of AD. *In vitro* analyzes were assayed and it was indicated that curcumin-loaded PLGA-PEG-B6 NPs had a narrowed diameter, increased cellular uptake, and good blood compatibility. In addition, these novel NPs could advance the spatial learning and memory capability of APP/PS1 mice compared with nonformulated curcumin and have promising properties in relieving b-cell amyloidosis and tauopathy with high bioavailability [123].

Huang et al. studied on other PLGA NPs containing S1 peptide as a A*β* generation inhibitor and curcumin. The iron-mimic cyclic peptide (CRT) is able to target the protein complex of transferrin and the transferrin receptor (TfR), and this conjugation provides increased BBB penetration, *in vivo* half-life, and therapeutic effects. According to this study, it was suggested that compared with other PLGA NPs, the CRT-conjugated NPs containing S1 peptide and curcumin showed increased in the BBB permeability of PLGA NPs and S1 peptide and curcumin played an important role in the treatment of AD mice by decreasing A*β* generation [124].

In a previous study, Meng et al. designed the curcumin-loaded low-density lipoprotein-mimic nanostructured lipid carrier (NLC) modified with lactoferrin (Lf) to target to the brain and evaluated its effect on the progression of the AD in rats. Authors used different levels of Lf for modification of NLC. *In vitro* characterization, uptake and cytotoxicity were measured in the brain capillary endothelial cells (BCECs). According to the release study, no significant difference was observed in terms of active content released from Lf-mimic NLC prepared with different levels of Lf. But, the increased uptake of Lf-mNLC by BCECs was found significantly compared with the NLC-modified reduced level of Lf, which reveals the process of Lf receptor-mediated endocytosis. A higher accumulation and controlling of progression of AD were exhibited with Lf-mNLC in the cortex and the third ventricle than those of NLC and it is possible to say that Lf-mNLC may be a novel modified nanocarrier for brain targeting [125].

Huo et al. designed Selenium curcumin nanoparticles encapsulated PLGA nanospheres and evaluated their effects in reducing Aβ aggregation in brain samples of mice with AD. Synthesized curcumin-loaded nanospheres were demonstrated that decreased the A*β* amount and improved the memory deficiency. Curcumin loaded Se-PLGA nanospheres were bound specifically to A*β* plaques and efficiently inhibited plaques. It was reported that these NP-encapsulated PLGA nanospheres could be targeted to amyloid plaques and could be an effective alternative to improve the therapeutic efficiency in AD lesions [126].

Tiwari and coworkers formulated a curcumin-encapsulated PLGA nanoparticle and evaluated its efficiency on neurogenesis and cognitive deficiencies. Authors focused on the activation of the Wnt/*β*-catenin pathway, nuclear translocation of *β*-catenin, and GSK-3*β* levels which are related to the regulation of neurogenesis by enhancement of expression of proneurogenic genes. These NPs were achieved that enhanced NSC proliferation and neuronal differentiation in vitro and reversed learning and memory impairments in amyloid beta-induced AD-like rat models as compared to nonformulated bulk curcumin [120].

As a new study, Wu conducted a comparative study by collecting all the data using the Morris water maze keyword to study AD in rodents by limiting the literature in general between years 2010 and 2021 and evaluating the spatial learning and memory of rodents, using the meta-analysis method. No statistically significant difference was found in their efficacy. It was concluded that both nanoparticles are equally effective in the treatment of AD [127].

In another study, research data showed that red blood cell (RBC) membrane-camouflaged human serum albumin nanoparticles bearing T807 and triphenylphosphine (TPP) molecules attached to the RBC membrane surface (T807/TPP-RBC-NPs) with curcumin as a model antioxidant nanosystem alleviated AD symptoms by reducing mitochondrial oxidative stress and suppressing neuronal death both *in vitro* and *in vivo* [128].

Curcumin-loaded solid lipid nanoparticles (SLNs) were determined by Kakkar et al. for brain delivery in rats orally. They suggested that the increased activity of acetylcholinesterase and two-fold concentration of curcumin in the brain were found compared to nonformulated curcumin when orally administered [129].

Another approach is magnetic NPs conjugated with curcumin, which specifically binds to amyloid plaques. Biocompatible curcumin-conjugated magnetic NPs coated with PEG-PLA block copolymer and PVP which had a mean diameter under 100 nm characterized their structure by spectroscopic methods. Curcumin-conjugated magnetic NPs were found nontoxic through the Madin-Darby canine kidney or differentiated human neuroblastoma cells. The apparent permeability coefficient of NPs was found to be 1.03 × 10–6 cm/s in an *in vitro* BBB model. *In vivo*, after injection of NPs, amyloid plaques were found in magnetic resonance imaging (MRI) of Tg2576 mouse brains. Immunohistochemically, examination of the mouse brains displayed that NPs showed high affinity to amyloid plaques and it was thought that NPs could be helpful for the diagnosis of AD using MRI [122].

Targeting the brain, improving pharmacokinetic and pharmacodynamic properties, and reducing side effects with the use of lipid-based nanocarriers are possible. Intranasal administration is a convenient way to cross the blood-brain barrier to target drugs towards the brain. By this noninvasive way, bypassing the BBB, presystemic metabolism may be eliminated [130]. At this point, in one study, curcumin-loaded AmyloLipid nanovesicles (ALNs) were fabricated to manage AD by inhibiting A*β* aggregation and A*β*-induced inflammation by nasal delivery. It was obtained after one hour of intranasal administration of the curcumin-loaded nanoparticles which showed 141.5 ± 55.9 ng/g brain levels and 11.9 ± 12.0 ng/mL plasma levels from in vivo study, and it can be said that a well-designed ALN containing curcumin given intranasally is a promising approach [131].

Chitosan-encapsulated PLGA core/shell NPs with curcumin, whose clinical applications are limited due to its low solubility and low bioavailability and first pass metabolism despite its strong therapeutic potential, were produced and evaluated with intranasal administration by Dhas and Metha. As a result of the release and permeability study, sustained release and improved permeability of the nasal mucosa were observed. In the *in vivo* study, the biodistribution of NPs was found to be higher in the brain following the intranasal route, demonstrating significant ROS scavenging effect on antioxidant activity. With all these data, it has been seen that NPs have potential to reduce oxidative stress in the brain for effective AD treatment [132].

Zhang et. al. produced chitosan-coated poly (lactic-co-glycolic acid) nanoparticles (CUR-CS-PLGA-NPs) and hydroxypropyl-*β*-cyclodextrin inclusion complexes of curcumin, which is difficult to administer due to its poor solubility and unstable structure, and compared them via intranasal administration. *In vitro* studies showed that inclusion complexes were more stable under physiological conditions than CS-PLGA-NPs and a higher cellular uptake of curcumin was obtained in SH-SY5Y cells. Both formulations were not cellular cytotoxic and showed a comparable antioxidant effect. *In vivo* pharmacokinetic studies showed that after the same dose intranasal administration, the AUC values of CUR in the plasma and brain of the inclusion complex which is both a simple method and structures suitable for scale-up in production were 2.57-fold and 1.12-fold higher than those of CS-PLGA-NPs, respectively [133]. Based on the data of this study and other studies with intranasal administration, it can be said that the intranasal route is an effective and good alternative for delivery of curcumin and other drugs/therapeutics to the brain or CNS.

Microemulsions (MEs) and nanoemulsions (NEs) are thermodynamically stable, and isotropically clear formulation consists of the oil phase and aqueous phase stabilized by the mixture of a surfactant and cosurfactant. Despite the advantages of ME/NE, few studies on using of NE studies were available for brain delivery. Sood et al. developed NE and mucoadhesive NE (mNE) systems to deliver curcumin for AD treatment. According to in vitro cytotoxicity and nasal ciliotoxicity studies, nontoxic and safe formulations were developed. The highest flux across sheep nasal mucosa was observed with mNEs compared to NE and curcumin solution, and it was suggested that poorly soluble curcumin could be given for nasal delivery for enhancement of brain uptake [134].

Again, curcumin/donepezil-loaded nanostructured lipid carriers (NCLs) were prepared and used with intranasal administration for delivery to the brain. A higher concentration of drugs in the brain was found with intranasal application compared to intravenous application. A mouse model was used, and it was obtained that the memory and learning were improved compared to the group treated with the nonformulated drug. Also, the improved ACh levels and decreased oxidation damage were found in the groups treated with NLCs [108].

In another published study, Mandal et al. aimed to design mucoadhesive ME gel (mMEg) to improve the brain uptake of curcumin through the intranasal route. After development of mMEg, characterization, stability, mucoadhesion, and nasociliotoxicity studies were performed and transparent, stable, and nonciliotoxic mMEg were obtained. Brain uptake of curcumin via nasal route was determined in rats. When curcumin mMEg was used, 11 times more curcumin uptake in the olfactory bulb was obtained compared to intravenous injection of curcumin solution. After nasal administration of curcumin mMEg, AUC ratios were significantly higher than those after intravenous administration of free curcumin [135].

In a novel study, Sharma reported the nanorobot drug delivery system to enhance bioavailability of curcumin during treatment of AD. The strategy of the design of the nanorobot drug delivery system with a specific target and pharmacokinetic formulation of the associated competing parallel reactions were examined. Chromophore was used as a sensor, and computer imaging and feedback control design may outcome in more bioavailability for curcumin therapeutic effect to treat AD [136].

Giacomeli et al. designed curcumin-loaded lipid-core nanocapsules (LNC) and evaluated the neuroprotective effects on a model of AD induced by intracerebroventricular injections of *β*-amyloid1-42 (A*β*1-42) peptide. Nonformulated curcumin (50 mg/kg, p.o.) and curcumin nanocapsules (10 or 1 mg/kg, p.o.) were given to aged female mice for 14 days after A*β*1-42 administration. Oral treatment for 14 days reduced both levels and protein expression of inflammatory cytokines which play a critical role in the neurodegeneration of AD. LNC shown neuroprotection effect significantly against A*β*1-42-induction which are associated with increased levels of cytokines. It was observed that the neuroprotective effect of curcumin increased with its nanoencapsulation and this nanocurcumin can be effective in the prevention and treatment of AD [137].

When we evaluate the data of all previous studies, it was seen that nanoformulations of curcumin helped in reducing the symptoms and treating AD. The advantages of nanoformulations were effective by increasing the low bioavailability, brain uptake, and therapeutic effect of curcumin. It can be said that these nanoformulations are an alternative which may be beneficial in the diagnosis and treatment of AD in the future. Also, it is seen that nanoformulations with different application routes help in overcoming the current bioavailability problem with the developing nanotechnology, taking into account patient compliance. However, the possible toxic effects of nanotechnology, which is developing and offers many possibilities, should not be ignored. Although cytotoxic and genotoxic side effects and allergic reactions have been reported in limited studies, extensive and long-term toxicity studies and data are needed.

## 10. Role of Curcumin in Sleep Disorders and Alzheimer's Disease: Insight from In Vitro and In Vivo Studies

Curcumin is predicted to be a neuroprotective agent, because of its potent antioxidant and anti-inflammatory activities, and therefore may be beneficial in the therapy of various neurological disorders and stress-related conditions such as AD.

Kumar et al. conducted research to investigate the function of nitric oxide in curcumin's preventive effects against 72-hour sleep deprivation behavioral alterations and oxidative damage in mice. Curcumin extract applied at 10 and 20 mg/kg (i.p) to mice for 5 days. Curcumin treatment restored depleted glutathione, catalase activity, and reduced lipid peroxidation and nitrite levels, preventing weight loss, decrease in locomotor activity, and anxiety-like effects across all behavioral paradigms. In addition, the inhibitor of nitric oxide synthase contributed in a meaningful way to the protective efficacy of curcumin [15].

Because alcoholism results in a variety of neuronal disorders such as the state of consciousness, sleep disorders, insomnia, dementia, and amnesia, the effects of curcumin on ethanol-induced 5-hydroxy-tryptamine (5-HT) and 5-hydroxy-indole acetic acid (5-HIAA) levels and rhythms on SCN and pineal were investigated. In the curcumin-treated group, after ethanol drinking, 0.002% (ad libitum) curcumin was given for 15 days. In curcumin-applied rats, a reduction in the amount of 5-HT and 5-HIAA was observed [138].

Noorafshan et al. conducted a research to analyze the potential effects of curcumin treatment on rapid eye movement sleep deprival (REM-SD) [16]. It was determined that the volume, cell number, reconstruction, object recognition time, and body weight were recovered in the REM − SD + curcumin group compared to the REM − SD + distilled water group. In a different study, Noorafshan et al. also examined the influence of curcumin on the medial prefrontal cortex (mPFC) of sleep deprivation in rats. Significant reduction in mPFC atrophic changes in cell loss and dendritic changes were surveyed in the SD animals after curcumin treatment [17].

In the study where the potency of curcumin on the intestinal microbiota of rats with interval sleep deprivation (ISD) was examined, 70 mg/kg curcumin was applied to the rats in the treatment group. According to the results of behavioral experiments, a meaningful progress was observed in the curcumin treatment group. This study proved that ISD not only leads to mental disorders in rats but also gives rise to alteration in *Escherichia coli*, *Bifidobacterium*, *Lactobacillus*, *Clostridium perfringens*, and *Bacteroides* and curcumin changed the microbiota imbalance [139].

Antioxidant levels, histopathological parameters, and the effect of curcumin on sleep deprivation liver injury in acute and chronic sleep-deprived rats were investigated by Saliha et al. According to the results of the study, pretreatment of curcumin showed a significant improvement in the acute sleep-deprived group, while positive changes were not significant in the chronic group. Biochemical findings are also supported by histopathological findings. The study reported that sleep deprivation can lead to oxidative stress damage in the liver, and dietary curcumin intake can increase the level of antioxidants in the body and reduce the risks caused by sleep deprivation [140].

More recently, Erfanizadeh et al. investigated the effect of chronic sleep deprivation on the superior cervical ganglion histomorphometrical changes and the ability of curcumin to prevent these changes. 36 male adult rats were randomly assigned into six groups including control, curcumin, sleep-deprived, and sleep-deprived + curcumin, grid floor, and grid floor + curcumin groups. One mL curcumin at the dose of 100 mg/kg/day (p.o.) curcumin applied to the curcumin groups for 21 days. With regard to stereological findings, CSD significantly decreased the volume of SCG and its total number of neurons and satellite glial cells when compared to the control group. Treatment of CSD with curcumin prevented reductions, and also, TUNEL assessment revealed significant apoptosis in SCG cells in the CSD group and curcumin significantly reduced this apoptosis. A decrease in apoptosis was observed in all curcumin-applied control groups. As a result, it has been reported that the harmful effects of sleep deprivation can be prevented by curcumin-administered orally [141].

Although there are few studies on sleep deprivation with curcumin, the ones that have been done show that when given during sleep deprivation, antioxidant effective curcumin prevents platform sleep deprivation neuron loss in the rat cortices. However, only one research used stereological methods to assess cell loss [141]. Studies reported that sleep deprivation increases oxidative stress or that curcumin decreased oxidative stress in animals protected from sleep loss neural injury.

A recent large-scale study finds that benzodiazepines, which are often used to treat anxiety and sleep problems, are associated with an increased risk for Alzheimer's disease in older people [142]; few studies reported effects on AD. In mouse cerebellar granule cells, ramelteon enhanced the neural content of BDNF. Therefore, it has been stated that if the application of ramelteon can regulate brain BDNF levels, it can be used as a possible therapeutic agent in neurodegenerative diseases such as AD [143].

A low dose of the tranquilizer diazepam decreased the breakdown of neurons, seen in the progress of Alzheimer's disease. Diazepam have been shown to increase spatial learning and memory retention and normalized protein concentrations associated with acetylcholine breakdown and GABA synthesis in rats induced with Alzheimer's disease [144].

Schetinger et al. studied the effect of new 1,5 benzodiazepines on acetylcholinesterase (AChE) and ATPDase (apyrase) activities in the adult rats' cerebral cortex. It was reported that 2-trichloromethyl-4-phenyl-3H-1,5-benzodiazepin and 2-trichloromethyl-4(p-methyl-phenyl)-3H-1,5-benzodiazepin significantly inhibited acetylcholinesterase activity between the range of 0.18–0.35 mM. Also at concentrations ranging from 0.063 to 0.25 mM, the 1,5 benzodiazepines inhibited ATP and ADP hydrolysis by synaptosomes from the cerebral cortex [145].

It is known that oxidative damage and inflammation takes place in age-correlated neurodegenerative ailments such as AD, while curcumin possess antioxidant and anti-inflammatory effects that provide significant preservation towards neurotoxic and genotoxic agents. Based on this, Frautschy et al. used 22-month-old Sprague-Dawley rats to compare the ability of ibuprofen and curcumin to protect against amyloid *β*-protein (Ap) detriment. Only dietary curcumin (2000 ppm) pressed oxidative damage and decrement in synaptophysin. Microgliosis in the cortical layers was lowered by both ibuprofen and curcumin. In the other group of the study, middle-aged female rats were treated with 500 ppm curcumin. The results showed that curcumin demonstrated the ability to prevent A*β*-infusion-initiated spatial memory deficits and postsynaptic density loss and decreased A*β* deposits [146].

Because metals induce amyloid-beta aggregation and toxicity, chelators, desferrioxamine, and clioquinol have significant effects against AD. Baum and Ng evaluated curcumin affinity for copper, zinc, and iron ions. It has been determined that curcumin binds redox-active metals iron and copper more easily than and redox-inactive zinc. As curcumin inhibits metal chelation, it has been clarified by the study that it can be protective against Abeta toxicity, and at the same time, it can depress inflammatory injury by preserving metal induction of NF-kappaB [147].

Under in vitro aggregation conditions, curcumin has been pointed to limited aggregation at a concentration of IC_50_: 0.8 *μ*M and disaggregated fibrillary Abeta40 IC_50_: 1 *μ*M. Also in the same study, curcumin was reported to be a preferable Abeta40 aggregation inhibitor than ibuprofen and naproxen and prevent Abeta42 oligomer occurrence at 0.1 to 1.0 *μ*M concentration [148].

Since AD is featured by the accumulation of amyloid beta in brain cells along with oxidative stress and inflammation, Balasubramanian investigated the efficacy of curcumin on amyloid beta plaques in his study. Balasubramanian reported that curcumin is easier to bind to amyloid beta, as it has appropriate charge and binding properties, while anti-inflammatory and antioxidant power is also dependent on electronic and structural features. Among the keto and enol forms, the enol form has been shown to have the capability to pass the blood-brain barrier and bind to amyloid beta and has all the characteristics for an optimum antioxidant [149].

Using multiple photon microscopy (MPM), Garcia-Alloza et al. have represented that curcumin passes the blood-brain barrier and label senile plaques and cerebrovascular amyloid angiopathy (CAA) in APPswe/PS1dE9 mice. Curcumin was given as i.v. from tail vein 7.5 mg/kg/day for 7 days. Curcumin removed and decreased substantial plaques that were monitored and also caused restricted but significant inversion of constitutional alterations in dystrophic dendrites. The outcomes of the study revealed that curcumin can be an effective preventive agent for AD-related oxidative stress, inflammation, and neurotoxicity [150].

Park et al. induced PC 12 cells with A*β* (25–35) to assess the effects of curcumin on neuroprotection. Before A*β* (25–35) induction, curcumin was treated at 10 *μ*g/mL concentration for 1 hour and it significantly inverted the activity of A*β* (25–35) by reducing oxidative stress and DNA detriment, besides intracellular calcium amounts and hyperphosphorylation of tau [151].

Intracerebroventricular streptozotocin (ICV–STZ)-induced rats were used to evaluate curcumin activity against cognitive deficiency and oxidative detriment exploiting passive avoidance and water maze tasks. During the three weeks, 80 mg/kg curcumin was applied. Cognitive deficiencies caused by ICV-STZ in the curcumin group improved significantly. Curcumin treatment clearly increased low levels of glutathione (GSH), glutathione peroxidase (GPx), and glutathione reductase (GR) in the hippocampus and cerebral cortex. Ishrat et al. reported that curcumin is efficient in preserving cognitive deficiency and may be useful in the treatment of Alzheimer's-type sporadic dementia [152].

In another study, the influence on cerebral blood flow (CBF), memory disruption, oxidative stress, and cholinergic dysfunction was investigated in memory disorder mice induced by IC-STZ with curcumin. Mice were given 10, 20, and 50 mg/kg, (p.o.) curcumin, for 21 days from the first application of STZ. In STZ-induced mice, curcumin increased the cerebral blood flow in a dose-dependent manner, thereby improving memory deterioration in both preservative and therapeutic ways [153].

Beta-amyloid (A*β*1-40) was used to induce the Alzheimer model in rats to evaluate the curcumin's effect on changes in the expression of the apoptosis-related genes Bax and Bcl-2 in the hippocampus, besides alterations in the spatial memory and cognitive function. Rats were given 300 mg/kg/day i.p. curcumin for seven days. In the curcumin-treated group, Bax expression was attenuated significantly, while Bcl-2 expression was augmented. The Morris water maze outcomes proved that curcumin clearly advanced rat spatial learning and memory functions by shortening the average escape latency via Bax and Bcl-2 modulation [154].

The influence of short- and long-time (6-week, 12-week) curcumin diet on hippocampal cellular proliferation and cognitive function were investigated in aged rats fed with a standard feed mixture of 480 mg/kg curcumin concentration. Researchers also explored the underlying mode of action that could connect curcumin treatment to enhanced cognition and neurogenesis by exon sequence analysis of cortical and hippocampal mRNA transcription. This study has been clarified with a transcriptional network interference of genes involved in neurotransmission, neuronal development, signal transduction, and metabolism after treatment with curcumin. Both short- and long-time curcumin treatments have improved the social recognition memory of aged rats in a meaningful way. Stronger improvement in spatial memory was observed after a long term of treatment, recommending that approximately 12 mg of long-term curcumin consumption per day may preserve or ease off cognitive function with aging. The mechanism underlying the effect of curcumin on learning and memory development in long-term treatment lies in promoting neurogenesis in the dentate gyrus, especially in aged rats [155].

Senescence-accelerated mouse prone 8 (SAMP8 mice) was used by Sun et al. to examine whether curcumin can cure cognitive defects. Curcumin was given intragastrically to mice at a daily dose of 20 and 50 mg/kg for 25 days. In this study, the Morris water maze test was used. Learning and memory of the mice were interpreted by examining the spatial memory, superoxide dismutase (SOD) activity, malondialdehyde (MDA) amount, expression of p-calcium/calmodulin-dependent kinase II (p-CaMKII), and p-N-methyl-d-aspartate receptor subunit 1 (p-NMDAR1) in the hippocampus. In both doses, curcumin significantly reduced escape delays and reduced the amount of MDA and in a dose-dependent manner enhanced the p-CaMKII and p-NMDAR1 expression [156].

Banji et al. used D-galactose to differentiate mitochondrial dynamics in rats and to generate oxidative stress in the apoptosis of neurons. Rats were given curcumin alone (50 mg/kg, p.o.), hesperidin alone (10 mg/kg, p.o.), and a combination of hesperidin and curcumin (10 mg/kg, 50 mg/kg, p.o.) one week before D-galactose treatment. In addition to behavioral studies, tricarboxylic acid cycle enzymes, mitochondrial complexes, oxidation of protein and lipid, and glutathione amounts in the mitochondrial fraction of brain were evaluated. At the same time, caspase-3 and cleaved caspase-3 were studied with Western blot analysis. The outcomes of the research referred that curcumin was found to be stronger than hesperidin in lowering lipid oxidation, proteins, cleaved caspase-3 expression, and amounts of mitochondrial enzymes. Cognitive development in combined therapy has been attributed to the preservation of morphological facets and the positive influence of the biochemical functions of neurons [157].

With the abovementioned studies, the potency of curcumin against AD has been proved with different mechanisms in vitro and in vivo. The efficacy of curcumin on AD is still under investigation and recent research has been summarized in the Table 1.

### 10.1. Clinical Studies on Curcumin

A 6-month curcumin clinical study was conducted to examine the effect of curcumin on biochemical and cognitive measures and safety on AD patients in China. Curcumin was applied in capsules or powder at 1 or 4 g. No dissimilarity was detected in the minimental condition examination. Also, the results indicate that there was no difference in serum A*β*1-40 levels between treatments and in the curcumin treatment group. AP aggregation in the brain was observed to decrease. Compared to bioavailability, it has been reported that capsules are better than powder and there is no difference between 1 and 4 g of curcumin in terms of metabolites [168] (Tables 2 and 3).

Curcumin, bisdemethoxycurcumin, and demethoxycurcumin mixture named Curcumin 3 Complex® was applied, 4 g/day during 24 weeks, to participants. No data on the effectiveness of the formulation in cognition and AP were found, and in CSF, tau levels and bioavailability in plasma were low [169].

Cox et al. conducted a study between 65- and 80-year-old adults. Treatment of 400 mg dose of Longvida® containing 80 mg curcumin was applied as acute chronic and acute on chronic. One hour after dose, curcumin application had a positive effect on working memory and continuous attention measurements. Researchers found that they saw a similar effect after chronic administration, and also, they stated that there were no similar results 3 hours after acute administration. In addition, in the chronic curcumin group, the mood developed; increased calmness and decreased prostration were observed [170].

Curcumin formulation BiocurcumaxTM has been studied for its prevention effect on cognitive decline in older adults in a double blind, randomized research. 1500 mg/d BiocurcumaxTM were given to participants for 12 months. In the study, no difference was observed between the groups for clinical and cognitive measurements [171].

Theracurmin®, containing 90 mg curcumin, was examined on 40 nondemented and 51–84-year-old participants. Formulation and placebo were applied twice a day for 18 months. The study's findings included a decrease in amyloid formation and tau accumalation, as well as better attention and memory. [172].

Although curcumin is effective against a large number of diseases, it has been shown to limit the therapeutic efficacy of poor bioavailability due to poor absorption, rapid metabolism, and rapid systemic elimination. As a result, numerous efforts have been made to improve the bioavailability of curcumin by modifying these properties. Adjuvants using to restrain curcumin's metabolic pathway is the most common strategy to enhance the curcumin's bioavailability. Also, other encouraging approaches to increase the bioavailability of curcumin in humans include nanoparticles, liposomes, phospholipid complexes, and structural changes [173].  Table 2 lists completed clinical trials with curcumin, while Table 3 lists unpublished clinical studies with curcumin.

### 10.2. Side Effects/Toxicity of Curcumin

In general, preclinical and clinical studies reported that curcumin has no significant side effect; limited numbers of study demonstrated minor adverse effects of curcumin at high doses. Curcumin is considered safe by the FDA. Due to curcumin's effect on cytochrome P450, glutathione-S-transferase, and UDP-glucuronosyltransferase, it has been shown that it affects the metabolism of varied drugs and reported to cause an increase in plasma concentration [174]. Also, in some studies, it has been clarified that in a dose-dependent manner, curcumin induces chromosomal changes and causes alterations of DNA in various mammalian cell lines [175, 176]. In a clinical trial of Sharma et al., nausea and diarrhea and increased levels of serum alkaline phosphatase and lactate dehydrogenase were observed over a 1–4 months, with the dose range of 0.45–3.6 g/day [177]. Hanai et al. also reported minor gastrointestinal side effects in ulcerative colitis patients [178]. On the other hand, many clinical studies with different time intervals and different doses reported no side effects and evaluated curcumin as safe [179–183].

## 11. Conclusion and Future Perspectives

Although *in vitro* and *in vivo* studies showed promising results, clinical studies demonstrated the low bioavailability of curcumin; hence, in low doses, no activity was seen in patients. In order to overcome this problem, new formulations with various administration routes should be developed. Furthermore, difficulties in diagnosis and even misdiagnosis of the neurological diseases, mainly AD, are still one of the major problems despite the technological improvements. For the most appropriate treatment regime and increased efficiency of the selected treatment, the diagnosis and mechanism of the disease must first be revealed correctly.

## Figures and Tables

**Table 1:** Summary of in vivo and in vitro studies in recent years.

Model	Duration of treatment	Dose	Effect	Reference
*Amyloid β-peptide (1-42) induced rats*	7 days	100 and (200 mg/kg, i.p. of curcumin	Curcumin treatment weakened cognitive impairments and an increase in BDNF levels in the hippocampus caused by AP1-42	[158]
*APPswe/PS1dE9 double transgenic mice*	6 months	500 ppm	Although FMeC1 (curcumin derivative) significantly reduced the cell toxicity of A*β*, both curcumin and FMeC1 extenuated the occurrence of A*β* aggregates	[159]
*Intracerebroventricular (ICV) streptozotocin-induced rats*	3 months	Curcumin (80 mg/kg/day, p.o.) and erythropoietin (500 IU/kg, i.p.)	Single curcumin supplementation could heal cognitive deficiency and reverse biochemical alterations	[160]
*18-month-old Sprague-Dawley rats*	60 days	(100 mg/kg/day, p.o.)	Curcumin enhanced memory and increased dendritic spine density and length in certain brain districts	[161]
*Middle-aged adult (male/female) rhesus monkeys*	8 months	500 mg per day dietary curcumin	Curcumin enhanced performance in repeated implementation of spatial working memory task	[162]
*6-Hydroxy dopamine-induced Wistar rat*	21 days	25 and 50 mg/kg, p.o.	Curcumin inhibited behavioral, neuroinflammatory, and neurochemical alterations and prevent the nigrostriatum's antioxidant potency	[163]
*Memory deficit p25 transgenic mouse*	12 weeks	Curcumin formulation, Longvida 0.8 g curcumin/kg	Curcumin intermediated suppression of neuroinflammation, attenuated the progression of p25-stimulated tau/amyloid pathology, and in order improved the p25-induced cognitive disorders	[164]
*HT22 murine hippocampal neuronal cells exposed to acrolein*	30 min	5 *μ*g/mL	Prevented AD-like pathologies, increased GSH and SOD, and attenuated MDA, A-disintegrin, metalloprotease, and also amyloid precursor protein, *β*-secretase, and receptor for advanced glycation end products modulating the BDNF/TrkB signaling	[165]
*APPsw transgenic mice*	6 months	Low dose 160 ppm, high dose 5000 ppm	160 ppm-dosed curcumin reduced CD33 and increased TREM2 expression and also augmented TyroBP, which controls AD-related neuroinflammatory gene network, besides CD68 and Arg1 phagocytosis markers	[166]
*B6C3-Tg (APPswePSEN1dE9)/Nju double transgenic mice*	3 months	Berberine: 100 mg/kg/day, curcumin: 200 mg/kg/day	The combined use of berberine and curcumin reduced the production of soluble amyloid-*β*-peptide (1–42) by showing synergistic effect. Decreased inflammatory responses and significant synergistic effects on oxidative stress were also observed in both the cortex and hippocampus of transgenic mice	[167]

**Table 2:** Completed clinical studies with curcumin.

Study (year)	Participants	Dose/duration	Study design
Baum et al., 2008, NCT00164749	36 participants with AD	1 g/4 g curcumin (capsules or as powder to be mixed with food)/placebo, 6 months	Double-blind, placebo controlled, randomized, pilot clinical trial
Ringman et al., 2012, NCT00099710	33 participants with wild to moderate AD	2 g/4 g curcumin C3complex/placebo, 6 months	A phase II, double-blind, placebo-controlled, randomized study
Paul H. Wand, NCT01716637	12 participants with AD	25 mg etanercept, administered weekly for 6 weeks; nutritional supplement (super biocurcumin) administered daily for 6 weeks	Open label, randomized, crossover, pilot study
Cox et al., 2015	60 healthy participants	400 mg Longvida/placebo, 1 h/3 h/4 weeks	Randomized, double-blind, placebo-controlled trial
Rainey Smith et al., 2016	96 healthy participants	1.5 g Biocurcumax/placebo, 12 months	Randomized, placebo-controlled, double-blind trial
Small et al., 2018	40 participants (nondemented)	90 mg of curcumin (Theracurmin) twice daily, 18 months	Randomized, double-blind, placebo-controlled trial

**Table 3:** Unpublished clinical studies with curcumin.

Study	Cohort	Intervention	Primary outcome	Main results
ClinicalTrials.gov, NCT01811381	80 participants with mild cognitive impairment (MCI)/subjective cognitive impairment	800 mg of curcumin (Longvida) in 4 capsules BID per day prior to meals, 12 months	Curcumin effects (first six-month period) or curcumin and aerobic yoga effects (second six-month period) on the changes in the levels of blood biomarkers for mild cognitive impairment relative to baseline or relative to placebo or nonaerobic yoga	Active, not recruiting
ClinicalTrials.gov, NCT03761381	20 participants with suspected dementia/AD	Oral curcumin supplement, 10 days	Curcumin will help improve protein detection with OCT and OCTA imaging taken twice, approximately 10 days apart	Recruiting
ClinicalTrials.gov, NCT04606420	100 participants with early AD	A low-fat (10–15%) whole-food vegan diet, high in complex carbs and low in refined carbs. Multivitamin, fish oil, curcumin, vitamin C, B12, CoQ10, lion's mane, probiotic, and magnesium (40 weeks)	Change from Baseline in Alzheimer's Disease Assessment Scale Cognitive Subscale (ADAS-Cog), Clinical Global Impression of Change (CGIC), Clinical Dementia Rating Scale Sum of Boxes (CDR-SOB) scores	Recruiting
ClinicalTrials.gov, NCT01001637	26 participants with AD	Curcumin formulation (Longvida), 2000 mg or 3000 mg daily BID, 2 months	Not mentioned	Unknown status

## Data Availability

The data used to support the findings of this study are all included and available within the article.
